# How do short-term associations between diet quality and metabolic risk vary with age?

**DOI:** 10.1007/s00394-020-02266-5

**Published:** 2020-05-13

**Authors:** Eleanor M. Winpenny, Esther M. F. van Sluijs, Nita G. Forouhi

**Affiliations:** MRC Epidemiology Unit, University of Cambridge School of Clinical Medicine, Institute of Metabolic Science, Cambridge Biomedical Campus, Box 285, Cambridge, CB2 0QQ UK

**Keywords:** DASH, Adolescent, Early adulthood, Diet quality, Metabolic syndrome, Cardiovascular risk

## Abstract

**Purpose:**

Poor diet quality is one of the key contributors to poor cardiovascular health and associated morbidity and mortality. This study aimed to assess how the short-term associations between diet quality and metabolic risk factors change with age.

**Methods:**

This longitudinal, observational study used data from the National Diet and Nutrition Survey (2008–2016) (*n* = 2024). Diet quality was measured using the Dietary Approaches to Stop Hypertension (DASH) index, fruit and vegetable (F&V) intake, and a F&V biomarker score. We assessed associations between measures of diet quality and a metabolic risk *z* score (generated from five metabolic risk factors) among those aged 11–60 years, and then tested effect modification by age group (adolescents 11–18 years, young adults 19–35 years, mid-aged adults 36–60 years).

**Results:**

Analysis across all age groups showed inverse associations between standardised DASH index and metabolic risk *z* score of − 0.19 (95% CI − 0.26, − 0.11). These associations were moderated by age group, with strong associations seen in mid-aged adults: − 0.27 (95% CI − 0.39, − 0.16), but associations were significantly attenuated in young adults [− 0.10 (95% CI − 0.22, 0.01)] and adolescents [0.03 (95% CI − 0.05, 0.11)]. Similar results were found for F&V intake and F&V biomarker score.

**Conclusions:**

Short-term associations between diet quality and metabolic risk are not consistent across adolescent and young adult age groups, suggesting that mechanisms by which diet impacts on metabolic risk may be acting differently in younger age groups compared to adults. Further research is warranted using longitudinal study designs and replication in different populations to understand changes in determinants of cardiometabolic health with age.

**Electronic supplementary material:**

The online version of this article (10.1007/s00394-020-02266-5) contains supplementary material, which is available to authorized users.

## Introduction

Cardiovascular disease is responsible for 26% of all deaths in the UK [1] and estimated to cost the UK economy £19 billion each year [1]. Poor diet quality is one of the key contributors to poor cardiovascular health and associated mortality and morbidity [2]. While the health consequences of a poor diet are primarily realised later in life, interventions to improve diet quality have been targeted across the life course, including, for example, school food interventions [3], and lifestyle interventions in young adults [4].

To develop appropriate dietary recommendations and interventions among children and young adults, there is a need for a better understanding of how diet at these ages contributes to health outcomes later in life. While cardiovascular disease and other related chronic diseases typically develop in older adults, the relationships between poor diet quality and adverse health outcomes are mediated by metabolic risk factors such as abdominal obesity, insulin resistance, hypertension and dyslipidemia. In combination, these risk factors have been referred to as the metabolic syndrome [5]. Metabolic syndrome emerges in childhood [5] when these metabolic risk factors are already strongly associated with the extent of atherosclerotic lesions [6]. However, there has so far been little comparative assessment of how the relationship between diet quality and metabolic health varies across the life course.

One method of quantifying overall diet quality is the DASH diet index, based on The Dietary Approaches to Stop Hypertension (DASH) Eating Plan. Randomised controlled trials of the DASH diet have shown reductions in cardiovascular disease risk factors, including systolic and diastolic blood pressure, and total and low-density lipoprotein (LDL) cholesterol among adult populations [7, 8]. Important components of the DASH diet are the high consumption of fruits, vegetables, whole grains, nuts and legumes, and low-fat dairy, and low consumption of red and processed meats, non-milk extrinsic sugars, and sodium. The majority of research linking the DASH diet index (which scores intake based on DASH diet components) to health outcomes has been conducted in adults, and performance of this index has been compared favourably to other diet quality indices in association with risk of chronic disease mortality [2]. While the DASH diet index has been judged appropriate to use in younger populations, data on associations with blood pressure and body mass index in these populations are mixed [9].

Nutritional biomarkers that reflect fruit and vegetable consumption have been used as a proxy measure for diet quality, with the advantage that these biomarkers can be measured objectively and thus avoid some of the potential biases and misreporting of self-reported dietary data. Different types of fruits and vegetables contain different levels of vitamin C and carotenoids (e.g. citrus fruits are high in vitamin C; apricots, spinach and carrots are good sources of beta-carotene, while kale, broccoli and green peas contain high levels of lutein). Levels measured in blood plasma have been shown to correlate with dietary intake [10], allowing these compounds to be used as concentration biomarkers of fruit and vegetable intake, with a composite score including a combination of vitamin C and carotenoids used to reflect intake across a wide range of fruits and vegetables [11, 12].

In this study, we assessed how the strength of the relationship between diet quality and metabolic risk factors varies across the age range from adolescents to mid-aged adults. We aimed to answer the research question: “How do short-term associations between diet quality and metabolic risk vary with age?” We made use of data from the UK-representative National Diet and Nutrition Survey, to study associations between self-reported and objective measures of diet quality, and a panel of metabolic risk factors.

## Subjects and methods

### Survey design and participants

Data were collected within the National Diet and Nutrition Survey (NDNS) Rolling Programme (2008–2016), an annual cross-sectional survey that assesses the diet, nutrient intake, and nutritional status of the general population of the UK. The NDNS aims to recruit 1000 participants each year, comprising an equal ratio of adults (aged 19 years and older) and children (aged 1.5–18 years). In each year of the survey, households were sampled from the UK Postcode Address File, a list of all addresses in the UK, with up to one adult and one child (1.5 years or older) from each household eligible for inclusion in the survey [13]. The survey was conducted in two stages: stage 1, an interviewer stage and stage 2, a nurse visit, typically conducted several months later. The interviewer stage comprised a food diary, a face-to-face Computer Assisted Personal Interview, height and weight measurements, questionnaires and a urine sample. Those who completed the interviewer stage were then invited to take part in a nurse visit, which included taking blood samples, further anthropometric measurements, and blood pressure measurement [13]. Written informed consent was obtained from each participant, or their parent/guardian if aged under 16 years. Ethical approval for the NDNS was obtained from the Oxfordshire A Research Ethics Committee and the Cambridge South NRES Committee (Ref. No. 13/EE/0016) and this research was conducted in accordance with the ethical standards laid down in the 1964 Declaration of Helsinki and its later amendments.

In this analysis, we used data on individuals assessed in years 1–8 of the NDNS Rolling Programme, who consented to a nurse visit and provided a blood sample. We included participants aged from 11 to 60 years, to comprise three age groups: adolescents (11–18 years), young adults (19–35 years) and mid-aged adults (36–60 years). The adolescent age group in this study started at age 11 years, as this is the youngest age at which data on waist circumference were available within NDNS. We defined early adulthood from age 19, in line with the adult definition within the NDNS dataset, up to age 35, which has been suggested as the end of young adulthood with regards to weight control [4]. We considered mid-aged adults to be from age 36 to age 60, excluding older adults who are more likely to experience chronic morbidity. Participants were excluded from the analyses if they reported taking anti-hypertensive or lipid-lowering medication, or reported implausible daily energy intake within the diet diary (see below).

### Dietary assessment and processing

Dietary assessment was conducted during stage 1 (the interviewer stage) of the data collection. Survey participants were asked to complete a food diary, covering four consecutive days, providing detailed descriptions of each food and drink item consumed, time of consumption and estimated amount, as described and validated previously [14, 15]. Three interview visits per participant were used to improve compliance in completing the food diary. A parent/carer was asked to complete the food diary for adolescents aged 11 years (as with younger children), with help from the child as appropriate. Adolescents aged 12 and older were asked to complete the food diary themselves, but details were confirmed with others where necessary. A food photograph atlas specifically designed for use with young people was used to assist with portion size estimation among those under 18 [14]. The protocol was designed so that all days of the week were equally represented across the sample. Data from completed food diaries were processed by trained coders, using the DINO (Data In, Nutrients Out) dietary assessment system [16]. Data files reported food group, nutrient, and energy intake data for each individual. Validation of energy intake data using doubly-labelled water showed underreporting of total energy intake on average.

Participants who had completed a food diary over three or more days were eligible for inclusion in the analysis. Individuals reporting consumption of less than 500 kcal/day or greater than 4800 kcal/day were excluded due to implausible energy intake, following an adaption of adult recommendations [17] to take into account the additional energy needs of growing adolescents [18].

### DASH diet index

Diet quality was assessed using a DASH index, based on the methodology used by Fung et al. [19] and adapted for use in NDNS [20, 21]. This index provides a diet quality score based on the relative intake of eight food and nutrient components (fruits, vegetables, whole grains, nuts and legumes, low-fat dairy, red and processed meats, non-milk extrinsic sugars, and sodium). Individual data were first adjusted for total energy intake using the residual method to account for misreporting and differences in energy intake with age [17]. For each DASH index component, participants were allocated into quintiles of intake and scored based on the quintile they populated. Five points were allocated to those in the most healthy quintile (highest consumption of fruit, vegetables, whole grains, nuts and legumes, low-fat dairy; lowest consumption of red and processed meats, non-milk extrinsic sugars, and sodium) and 1 point allocated to those in the least healthy quintile. The resulting scores were summed across the eight index components, to give a total score out of 40.

### Fruit and vegetable intake

Data on fruit and vegetable intake were processed as part of the DASH diet index, and adjusted for total energy intake using the residual method, as described above. Data on fruit and vegetable intake, therefore, represent the average daily intake of fruits and vegetables in grams, adjusted to a total energy intake of 2000 kcal.

### Fruit and vegetable biomarker score

A nutritional biomarker for fruit and vegetable (F&V) intake provides an alternative, more objective measure of diet quality. We used a composite biomarker score, based on plasma vitamin C and two carotenoids; beta-carotene and lutein. Fasted blood samples were collected and processed by the Medical Research Council Elsie Widdowson Laboratory. Further details of the assays used to quantify vitamin C, beta-carotene and lutein levels and quality control data are detailed in NDNS Appendix Q [22]. The biomarker score was calculated by summing the standardised values for blood levels of vitamin C, beta-carotene and lutein, and then re-standardised. This biomarker score has been previously shown to provide a good representation of F&V intake and had a strong inverse association with incident diabetes [11].

### Metabolic risk factors

Metabolic syndrome and the component metabolic risk factors have been conceptualised and calculated in several different ways [5]. It has been argued that the use of a continuous metabolic risk score is statistically more sensitive and robust than defining metabolic syndrome based on predefined cut-offs [23]. Moreover, given the differences in reference values for metabolic risk scores in children compared to adults [24], use of a continuous score, standardised for age, facilitates comparison across different age groups. In this study, we assessed metabolic health using a metabolic risk *z* score (MetZscore), based on the metabolic risk factors included in the definition of metabolic syndrome from the National Cholesterol Education Program—Adult Treatment Program III (NCEP-ATPIII): waist circumference, blood pressure, serum triglycerides, serum high-density lipoprotein (HDL) cholesterol, and fasting plasma glucose [5].

Waist circumference was measured at the midpoint between the iliac crest and the lower rib (to the nearest 0·1 cm) using a tape measure. Systolic and diastolic blood pressures were measured in a seated position after participants had not eaten, consumed alcohol, exercised, or smoked in the preceding 30 min. Fasted blood samples were processed to measure serum triglycerides, serum high-density lipoprotein (HDL) cholesterol, and plasma glucose. Details of each assay and quality control data are detailed in NDNS Appendix Q [22].

The MetZscore was calculated based on a method previously used in paediatric research, first standardising the individual metabolic risk factors and then summing these standardised measures to create an overall score [23]. Standardisation of risk factors was achieved by regressing each risk factor on selected demographic variables: age, sex and ethnicity. Due to non-linearity of associations between metabolic outcomes and age, a restricted cubic spline regression was applied to the weighted sample, with knots at age 15, 20, 30, 40 and 50 years, to ensure appropriate adjustment for age-related variation. The resulting residuals were standardised to give a mean of 0 and standard deviation of 1. Standardised residuals of systolic and diastolic blood pressure were averaged to give a single value of mid-blood pressure [25]. Standardised residuals for HDL cholesterol were multiplied by − 1 to account for the inverse relationship of HDL cholesterol with metabolic risk. The standardised residuals for the individual risk factors were then summed to create the MetZscore, which was again standardised to mean 0 and standard deviation of 1. A higher score is indicative of a less favourable metabolic syndrome profile.

### Covariates and other descriptive variables

Age, sex, ethnicity, smoking, hours of sleep and long-term health conditions of the participant were self-reported. Ethnicity was classified according to five groups (white, mixed, black, Asian and other). Socio-economic class (SEC) of the household reference person was reported by the household reference person (defined as the householder with the highest income) and classified into four groups: Managerial and professional occupations, Intermediate occupations, Routine and manual occupations, and Never worked or other. Smoking was categorised into three categories: ‘current smoker’, ‘ex-regular smoker’ and ‘never regular smoker’. Height and weight were measured at the first interviewer visit, using a portable stadiometer, measuring to the nearest 0.1 cm and weighing scales, measuring to the nearest 0.1 kg. BMI was calculated as [weight (kg)/height (m^2^)], and converted to BMI *z* scores using the UK 1990 BMI reference curves [26]. Adult BMI was also expressed as a *z* score, compared to the oldest age for which a reference standard is available (age 23 years), as has been done previously [27]. Use of dietary supplements was based on reporting taking any type of dietary supplement (including vitamins, minerals and non-nutrient supplements) in the food diary. We tested inclusion of time spent in moderate/vigorous physical activity (available for those aged 16 + years only) as a covariate in our analyses, but found no evidence of confounding of associations by physical activity, so physical activity was not included as a covariate.

### Statistical analysis

All analyses were performed using STATA version 14 (StataCorp., College Station, TX, USA). Weights were applied to the NDNS dataset to account for complex survey sampling design and response bias. We used the blood sample weights provided with the dataset which additionally accounted for non-response by individuals to provide a blood sample.

For descriptive analyses, sociodemographic variables and dietary variables were summarised overall, and by age group. Linear regression was used to assess differences in dietary and metabolic variables between age groups. We assessed correlations between the three diet variables using the Pearson correlation coefficient, to examine how closely these variables were related.

Missing data was below 2%, except for F&V biomarkers (5% missing) and hours of sleep (20% missing). Multiple imputations by chained equations were used to impute missing data for covariates, under the missing at random assumption, following recommendations from White et al. [28]. Twenty imputed datasets were generated using the Stata command ice. Each dataset was analysed separately and results combined using Rubin’s rule [28].

Linear regression models were used to assess associations between standardised dietary exposures and metabolic outcomes. Model 1 was unadjusted. Model 2 was adjusted for socioeconomic class (SEC), smoking status, hours of sleep, taking dietary supplements and having a long-term health condition. Models were not adjusted for physical activity or alcohol intake as these were found not to be associated with the outcome variable after conditioning on diet quality. Further adjustment of the regression models for age, sex or ethnicity was not necessary as these were accounted for as part of the method for generating MetZscores. Similarly, adjustment for total energy intake was incorporated into the DASH index and F&V intake (see above) so further adjustment for total energy intake was not necessary. In model 3, an interaction term between age category and the diet variable was added to the adjusted model, to test for the moderation of the diet quality-MetZscore associations by age group. Results from these interaction analyses are presented as the overall beta coefficient for each age group, taking into account the association of each age group and interaction terms.

Model 4 was based on model 3 but included BMI *z* score as an additional confounder. BMI *z* score is hypothesized to act as a negative confounder of associations between self-reported diet and metabolic outcomes, where higher BMI may cause improved diet quality or increased misreporting [29], but as a positive confounder of associations between F&V biomarker score and metabolic outcomes, where BMI is known to be inversely associated with plasma concentrations of carotenoids [30, 31]. In both cases, BMI may additionally act as a mediator of the diet-outcome relationships.

Linear regression models were used to assess associations between dietary exposures and individual metabolic outcomes, standardised by age, sex and ethnicity as described above. Covariates and interactions were included as for model 3. Given the evidence of confounding by BMI in nutritional biomarker analyses, but not self-reported diet analyses, we include BMI *z* score as a covariate in our analysis of associations of nutritional biomarkers, but not self-reported diet, with individual metabolic outcomes. Results are presented as the change in *z* score of each outcome for one standard deviation change in the dietary exposure.

## Results

Of 6,932 participants aged between 11 and 60 years who took part in years 1–8 of the NDNS Rolling Programme, 2207 had complete data on metabolic risk. A further 177 individuals were excluded because they were taking anti-hypertensive or lipid-lowering medications, and another six based on reporting of unrealistic energy intakes. This left 2024 individuals in the analysis (for a participant flow chart see Supplemental Fig. 1).

Descriptive data on the study population are shown in Table 1. As shown by differences in DASH index, F&V intake and F&V biomarker scores, diet quality was lower among adolescents and young adults compared to mid-aged adults. Pearson correlation coefficients were used to assess how closely the dietary exposure measures were related: DASH index was highly correlated with self-reported F&V intake (*r* = 0.69). Self-reported F&V intake was moderately correlated with F&V biomarker score (*r* = 0.44). The DASH index was moderately correlated with F&V biomarker score (*r* = 0.39). *p* values for all correlations were *p* < 0.001.

**Table 1:** Characteristics of included study participants, by age group

	All included ages (11–60 years) (*n* = 2024)	Adolescents (11–18 years) (*n* = 623)	Young adults (19–35 years) (*n* = 443)	Mid-aged adults (36–60 years) (*n* = 958)
Sex (%)				
Female	51.8	47.9	50.9	53.6
Socio-economic classification of household reference person (%)				
Managerial and professional occupations	47.8	39.8	38.5	56.4
Intermediate occupations	20.1	22.2	21.2	18.8
Routine and manual occupations	27.9	31.2	35.2	22.0
Never worked and other	4.2	6.8	5.2	2.8
Ethnic group (%)				
White	87.4	86.7	83.9	90.1
Mixed ethnic group	2.5	3.4	3.9	1.2
Black or Black British	2.7	1.7	3.8	2.1
Asian or Asian British	6.0	6.8	7.4	4.9
Any other group	1.4	1.4	1.1	1.6
Smoking status (%)				
Current smoker	15.3	7.6	21.8	13.2
Hours of sleep				
Mean (SE)	7.4 (0.04)	8.9 (0.09)	7.6 (0.07)	7.1 (0.05)
BMI *z* score				
Mean (SE)	0.93 (0.04)	0.60 (0.06)	0.71 (0.09)	1.17 (0.05)
Dietary supplements (%)				
Taking supplements	21.4	11.0	18.0	26.9
Long-term health condition (%)				
Yes	22.6	10.9	19.3	28.4
DASH index (range 0–40)				
Mean (SE)	23.8 (0.18)	21.4 (0.25)***	22.9 (0.35)***	25.2 (0.23)
F&V intake (portions /2000 kcal total energy intake)				
Mean (SE)	3.49 (0.07)	2.40 (0.07)***	3.29 (0.12) ***	3.95 (0.09)
F&V biomarker score (standardised)				
Mean (SE)	0.00 (0.04)	− 0.13 (0.04)***	− 0.12 (0.07)*	0.12 (0.05)

National Diet and Nutrition Survey Rolling Programme, years 1–8

Numbers of individuals in each age category refer to unweighted numbers. The remaining table statistics are weighted to account for sample design and response biases

*DASH* dietary approaches to stop hypertension, *F&V* fruit and vegetable

Statistical tests for differences in diet and metabolic variables, by age group: ***p < 0.001, **p < 0.01, *p < 0.05, reference category: mid-aged adults.

### Associations of diet quality, F&V intake and F&V biomarkers with MetZscore

As shown in Table 2, there were inverse associations between each measure of diet quality (DASH score, self-reported F&V intake, F&V biomarker score) and MetZscore in the full sample (11–60 years) (Table 2). For example, each standard deviation (SD) increase in DASH score was associated with a − 0.19 SD (95% CI − 0.26, − 0.11) reduction in MetZscore. Inclusion of interactions between age and diet quality measures (model 3) showed that associations between diet quality and MetZscore differed by age group, with the strength of associations increasing with age across all measures of diet quality. For the DASH score, associations with MetZscore ranged from 0.03 SD (95% CI − 0.05, 0.11) in adolescents, to − 0.10 SD (95% CI − 0.22, 0.01) in young adults and − 0.27 SD (95% CI − 0.39, − 0.16) in mid-aged adults. Results from model 4, additionally adjusted for BMI *z* score, were attenuated compared to model 3, particularly among F&V biomarker associations. Adjustment for BMI resulted in associations which were more similar across the different diet exposure measures than unadjusted models.

**Table 2:** Cross-sectional associations between standardised dietary variables (DASH score, F&V intake, F&V biomarker score) and MetZscore, by age group

	Model 1, unadjusted		Model 2, adjusted		Model 3, adjusted and with interaction with age group		Model 4, with additional adjustment for BMI	
	*β*	95% CI	*β*	95% CI	*β*	95% CI	*β*	95% CI
DASH score								
All ages	− 0.21	− 0.28, − 0.15	− 0.19	− 0.26, − 0.11				
Age 11–18					0.03^$$$^	− 0.05, 0.11	0.03^$$$^	− 0.04, 0.10
Age 19–35					− 0.10^$^	− 0.22, 0.01	− 0.10	− 0.18, − 0.02
Age 36–60					− 0.27	− 0.39, − 0.16	− 0.17	− 0.27, − 0.08
F&V intake								
All ages	− 0.19	− 0.25, − 0.12	− 0.16	− 0.23, − 0.10				
Age 11–18					0.03^$$^	− 0.07, 0.12	0.05^$$^	− 0.02, 0.13
Age 19–35					− 0.11	− 0.20, 0.01	− 0.09	− 0.16, − 0.02
Age 36–60					− 0.21	− 0.31, − 0.10	− 0.14	− 0.22, − 0.05
F&V biomarker score								
All ages	− 0.33	− 0.39, − 0.27	− 0.31	− 0.37, − 0.25				
Age 11–18					− 0.16^$$$^	− 0.26, − 0.06	− 0.03^$$$^	− 0.13, 0.07
Age 19–35					− 0.26	− 0.36, − 0.16	− 0.13	− 0.20, − 0.05
Age 36–60					− 0.36	− 0.44, − 0.28	− 0.22	− 0.29, − 0.15

National Diet and Nutrition Survey Rolling Programme, years 1–8

All variables are standardised and beta coefficients are presented in units of standard deviations. Models 2 and 3 were adjusted for social class, smoking status, hours of sleep, taking dietary supplements and having a long-term health condition. Model 3 presents estimates for each age group, derived from a model including interaction between exposure and age group. Model 4 is as model 3, with additional inclusion of BMI *z* score as a covariate

*DASH* dietary approaches to stop hypertension, *F&V* fruit and vegetable

Interaction with age group: $ indicates significant interaction between diet exposure and age category, compared to the reference age category (36–60 years). ^$$$^*p* < 0.001, ^$$^*p* < 0.01, ^$^*p* < 0.05

Table 3 shows the associations between diet quality and the individual (standardised) components of the MetZscore. Positive associations were seen between HDL cholesterol and all metabolic outcomes, with inverse associations were seen for the remaining metabolic risk factors. Overall, the strongest associations were seen in the oldest age group across all diet quality measures, with associations attenuated in adolescent and young adult age groups. The only significant association seen among adolescents was the positive association of HDL cholesterol with the F&V biomarker score. Among young adults, HDL cholesterol was positively associated and waist circumference inversely associated with F&V biomarker score. Table 3 includes adjustment for BMI *z* score as a confounder in the F&V biomarker models only; a sensitivity analysis which included adjustment for BMI *z* score in associations between self-reported diet and metabolic outcomes showed very little alteration in findings, except for waist circumference (see Supplemental Table 1 of the Online Supporting Material for further details).

**Table 3:** Cross-sectional associations between standardised dietary variables (DASH score, F&V intake, F&V biomarker score) and standardised metabolic outcomes, by age group

	Waist circumference *z* score, *β* (95% CI)	Mid-BP *z* score, *β* (95% CI)	Glucose *z* score, *β* (95% CI)	HDL cholesterol *z* score, *β* (95% CI)	Triglycerides *z* score, *β* (95% CI)
DASH index					
All ages (11–60 years)	− 0.13 (− 0.20, − 0.07)	− 0.07 (− 0.14, − 0.01)	− 0.13 (− 0.21, − 0.04)	0.11 (0.03, 0.19)	− 0.12 (− 0.19, − 0.06)
Age 11–18	0.02^$$^ (− 0.08, 0.12)	− 0.02 (− 0.10, 0.06)	0.03^$^ (− 0.03, 0.09)	− 0.04^$^ (− 0.13, 0.04)	0.00^$$^ (− 0.07, 0.07)
Age 19–35	− 0.06 (− 0.20, 0.09)	− 0.05 (− 0.15, 0.05)	− 0.12 (− 0.23, 0.00)	0.08 (− 0.05, 0.20)	− 0.01^$$^ (− 0.10, 0.08)
Age 36–60 (ref for interaction)	− 0.19 (− 0.27, − 0.11)	− 0.12 (− 0.21, − 0.03)	− 0.14 (− 0.28, − 0.01)	0.15 (0.02, 0.28)	− 0.23 (− 0.36, − 0.11)
F&V intake					
All ages (11–60 years)	− 0.11 (− 0.17, − 0.05)	− 0.08 (− 0.14, − 0.02)	− 0.10 (− 0.19, − 0.01)	0.10 (0.04, 0.17)	− 0.11 (− 0.17, − 0.04)
Age 11–18	− 0.04 (− 0.15, 0.07)	− 0.00 (− 0.11, 0.10)	0.02 (− 0.06, 0.10)	− 0.03^$^ (− 0.14, 0.08)	0.07^$$^ (− 0.01, 0.15)
Age 19–35	− 0.05 (− 0.17, 0.08)	− 0.06 (− 0.17, 0.04)	− 0.12 (− 0.21, − 0.02)	0.06 (− 0.04, 0.16)	− 0.04 (− 0.11, 0.03)
Age 36–60 (ref for interaction)	− 0.13 (− 0.21, − 0.05)	− 0.12 (− 0.21, − 0.03)	− 0.09 (− 0.24, 0.06)	0.12 (0.02, 0.22)	− 0.17 (− 0.28, − 0.05)
F&V biomarker score					
All ages	− 0.09 (− 0.13, − 0.04)	− 0.12 (− 0.18, − 0.06)	− 0.07 (− 0.12, − 0.01)	0.23 (0.16, 0.29)	− 0.08 (− 0.15, − 0.01)
Age 11–18	− 0.00 (− 0.10, 0.09)	− 0.04 (− 0.14, 0.06)	0.06^$^ (− 0.04, 0.16)	0.14 (0.04, 0.25)	0.03^$$^ (− 0.05, 0.10)
Age 19–35	− 0.09 (− 0.17, − 0.01)	− 0.10 (− 0.20, 0.01)	− 0.02 (− 0.09, 0.05)	0.19 (0.07, 0.31)	0.00^$^ (− 0.09, 0.10)
Age 36–60 (ref for interaction)	− 0.05 (− 0.10, − 0.01)	− 0.14 (− 0.22, − 0.06)	− 0.10 (− 0.18, − 0.01)	0.25 (0.17, 0.33)	− 0.14 (− 0.24, − 0.03)

National Diet and Nutrition Survey Rolling Programme, years 1 to 8

Mid-BP is the average of systolic and diastolic BP. Models were adjusted for social class, smoking status, hours of sleep, taking dietary supplements and having a long-term health condition, and age-group specific covariates were derived from a model including interaction between exposure and age group. The F&V biomarker models were additionally adjusted for BMI *z* score

Interactions with age group: $ indicates significant interaction between diet exposure and age category, compared to the reference age category (36–60 years). ^$$$^*p* < 0.001, ^$$^*p* < 0.01, ^$^*p* < 0.05

## Discussion

In these analyses, we have shown that while higher diet quality was associated with lower metabolic risk in analysis of the whole population (aged 11–60 years), these relationships were not consistent across different age groups. When adolescents, young adults and mid-aged adults were considered separately, the strength of the association between diet quality and metabolic risk increased with age, with little or no association seen in adolescents, and a smaller association in young adults compared to mid-aged adults. Similarly, associations between diet quality and individual metabolic outcomes were typically only statistically significant in the mid-aged adult population. While stronger associations in adolescent and young adult age groups were initially seen with dietary biomarkers, these associations were attenuated after adjustment for BMI, suggesting confounding of biomarker associations by BMI, as has been reported previously [10]. Adjusted results were consistent across the three measures of dietary quality. These findings derived from analysis of a contemporary, UK-representative population, suggesting that in the UK, the diet quality of adolescents is not associated with their metabolic health over a short time frame (several months).

### Strengths and limitations of the study

Strengths of this study include its use of nationally representative data from eight consecutive years of the NDNS. The NDNS is often referred to as a cross-sectional study, which may be regarded as a limitation. However, data collection each year is completed in two stages, an interview stage (which includes the diet diary) and a nurse visit (including measurement of metabolic risk factors), which typically takes place several months later. Therefore, for analysis of relationships between diet and metabolic outcomes, this may be considered short-term longitudinal data with the opportunity for a diet to causally influence metabolic outcomes. Data were not available to adjust for the outcome measures at baseline, however, reverse causality is unlikely, given that study participants are unlikely to be aware of their metabolic health (with the exception of waist circumference and BMI), so this would not be expected to influence their diet. The associations remained after adjustment for baseline BMI. Since this is an observational study, there is always the opportunity for residual confounding, however, we have considered the inclusion of a wide range of covariates, and included these in our models where appropriate [32].

A further strength is the use of both self-reported diet data from diet diaries, considered to be one of the more robust methods of dietary assessment in free-living individuals, including adolescents [33], and objective data on nutritional biomarkers. We assessed diet quality using a well-recognized measure of diet quality, the DASH index. While there are a number of different options for assessing diet quality, the DASH index is reported to perform well in comparison with other diet quality scores at predicting health outcomes [34] and has been suggested to be particularly appropriate for use in adolescents given its inclusion of low-fat dairy as a positive component of diet quality [35]. We also assessed diet quality using nutritional biomarkers (vitamin C, beta-carotene and lutein). These biomarkers are objective indicators of fruit and vegetable intake, and use of a combination of different biomarkers has been shown to better predict fruit and vegetable intake than single biomarkers [10]. Biomarkers are not a direct measure of nutritional intake, also reflecting nutrient bioavailability and absorption, with differences in correlation between intake and plasma levels of biomarkers reported by sex, smoking and BMI [10]. Indeed, adjustment of our models for BMI *z* score made our findings based on self-reported diet and biomarker measurement more comparable, suggesting that biomarker results were confounded by BMI [30, 31]. Use of both high-quality self-reported diet and nutritional biomarkers enabled triangulation of approaches with different biases, and similar results across these different approaches strengthens our conclusions [36].

The use of a metabolic risk *z* score provides a single metabolic outcome which can be applied across the age range of interest. Standardisation of the *z* score components by age allows comparison of associations across the age range, taking into account the variation in mean levels of the included risk factors by age. A limitation of this method is that the *z* score is population-specific and does not allow comparison against reference values for metabolic risk.

### Comparison with previous evidence

A number of studies have examined associations between DASH scores and metabolic outcomes in age-based sub-populations. There is mixed evidence on associations between DASH diet quality measures and metabolic outcomes in adolescents. A recent systematic review identified five studies which assessed associations between the DASH diet, adiposity and blood pressure in healthy adolescents [9]. This included three cross-sectional studies, of which only one found clear associations between DASH diet and adiposity-related outcomes while a second found associations with systolic blood pressure but not diastolic blood pressure, body weight or waist circumference. Meanwhile, a recent meta-analysis of population-based observational studies found an overall association between F&V consumption and decreased risk of metabolic syndrome, which remained significant when restricted to the three cross-sectional studies which focused on adolescents [37]. Our own findings add to this evidence base, suggesting no associations between diet quality and MetZScore in adolescents, and reduced associations in young adults compared with mid-aged adults. Explanation for differences between studies may include the differences in the underlying populations where the studies were conducted, or differences in study design, for example differences between continuous assessment of metabolic risk *z* score compared to a cut-off for metabolic syndrome. Of the three studies contributing to the meta-analysis, two derived from the US, and one from Iran, which may represent populations with different health profiles from the UK adolescent population.

Other studies which have characterised diet quality in different ways have also in some cases shown associations with cardiometabolic health in children and adolescents. A review of dietary patterns and metabolic risk factors supported an association between unhealthy dietary patterns (high in energy-dense, high-fat and low-fibre foods) and metabolic risk, but with mixed and sometimes unexpected findings for healthy dietary patterns [38]. It could be that measures of unhealthy food intake are more consistently predictive of cardiometabolic health in young people than scores of overall diet quality such as the DASH diet index. Future studies could assess whether the associations between unhealthy dietary patterns and metabolic risk show similar moderation by age as we have seen in this study.

### Implications, possible mechanisms and questions for further research

This is the first study to our knowledge to examine how associations between diet quality and metabolic outcomes are moderated by age, suggesting that short-term associations between diet quality and metabolic health are less strong in adolescence and early adulthood compared with mid-aged adults.

One possible mechanism for the observed differences by age is that the effects of diet quality on metabolic outcomes become more pronounced in the presence of age-related dysregulation of metabolic and immune homeostasis [39]. Prevalence of overweight and obesity rises through early adulthood [40], and markers of the low-grade, chronic inflammation, considered to be an important factor in the pathogenesis of atherosclerosis and cardiometabolic disease [41], also increase with age [39]. While the mechanisms of action of dietary components on metabolic risk factors are complex, pathways such as the role of dietary antioxidants in reducing oxidative stress and inflammation [42] are likely to become more important in an inflammatory environment [43]. A further possible contributor to this complexity is the role of physical activity, which although not identified as a confounder of these analyses, has previously been shown to attenuate metabolic risk in overweight and obese adolescents [44]. Further work will be needed to confirm and to understand in more detail the processes underlying the age-related moderation of diet-metabolic health relationships reported here.

Our findings might initially be interpreted to suggest that adolescents and young adults do not need to be concerned about their diet. However, as discussed above, further analysis is needed considering different aspects of diet, such as unhealthy dietary patterns. Furthermore, it may be that diet quality in adolescence can have an impact on metabolic health over a longer time frame, and a small number of cohort studies have suggested longer-term associations between diet quality and metabolic health outcomes [9]. To date, there have been no life course studies of diet explicitly investigating the impact of diet quality during different periods of life on health outcomes [45]. Good quality data and careful analysis will be needed to separate true longer-term effects of adolescent diet from the impact of tracking of dietary habits over time. Such research will contribute to a better understanding of the appropriateness of applying measures of diet quality and dietary guidelines developed for adult populations to adolescents and young adults. However, we should also note that adolescence and early adulthood are periods of lifestyle change and formation of adult behaviour patterns [46], and as such supporting individuals to adopt healthy diets at this stage in their lives may allow persistence of these habits and support long-term health in later life.

## Conclusions

In these analyses, we have shown that while higher diet quality shows short-term associations with lower metabolic risk in mid-aged adults, these relationships are not consistent across younger age groups. This suggests that the mechanisms by which diet impacts on metabolic risk may be acting differently in younger age groups compared to adults, or may operate over a longer time frame. Further research with longitudinal follow-up and replication in different populations will be required to further understand changes in determinants of cardiometabolic health by age.

## Electronic supplementary material

Below is the link to the electronic supplementary material.

Supplementary file1 (DOCX 44 kb)
