# Supplementary material for: How do short-term associations between diet quality and metabolic risk vary with age?
Source: Eur J Nutr. 2020 May 13;60(1):517–27. doi: 10.1007/s00394-020-02266-5 (PMC7867538; doi:10.1007/s00394-020-02266-5)
Supplement: Supplementary file 1 — Supplementary file1 (DOCX 44 kb) [file 394_2020_2266_MOESM1_ESM.docx]

**Supplemental Figure 1:** Participant flow chart

**
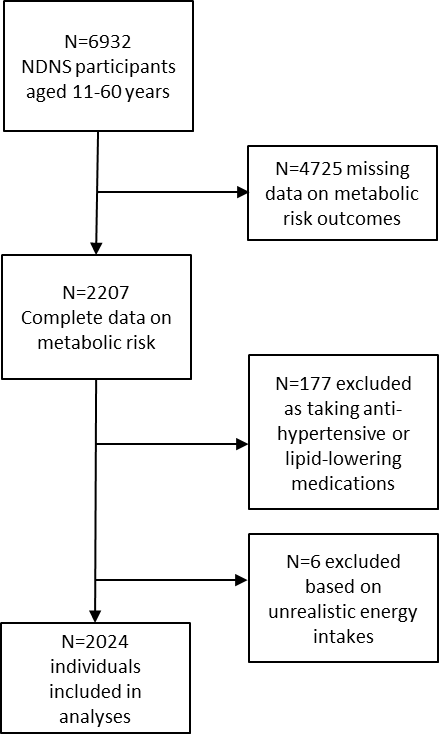
**

**Supplemental Table 1:** Cross-sectional associations between standardised dietary and standardised metabolic outcomes, by age group. National Diet and Nutrition Survey Rolling Programme, years 1 to 8.

|  | | Waist circumference | Blood pressure | Glucose | HDL cholesterol | Triglycerides |
| --- | --- | --- | --- | --- | --- | --- |
| DASH index | | | | | | |
| All ages  (11-60 years) | Model 3 | -0.13  (-0.20, -0.07) | -0.07  (-0.14, -0.01) | -0.13  (-0.21, -0.04) | 0.11  (0.03, 0.19) | -0.12  (-0.19, -0.06) |
|  | Model 4 | -0.09  (-0.13, -0.05) | -0.06  (-0.12, -0.00) | -0.12 (-0.20, -0.04) | 0.09 (0.02, 0.17) | -0.11 (-0.18, -0.05) |
| Age 11-18 | Model 3 | 0.02^$$^  (-0.08, 0.12) | -0.02  (-0.10, 0.06) | 0.03^$^  (-0.03, 0.09) | -0.04^$^  (-0.13, 0.04) | 0.00^$$^  (-0.07, 0.07) |
|  | Model 4 | 0.03  (-0.04, 0.10) | -0.02  (-0.09, 0.06) | 0.03^$^ (-0.03, 0.09 | -0.05 (-0.13, 0.04) | 0.00^$$^  (-0.06, 0.07) |
| Age 19-35 | Model 3 | -0.06  (-0.20, 0.09) | -0.05  (-0.15, 0.05) | -0.12  (-0.23, 0.00) | 0.08  (-0.05, 0.20) | -0.01^$$^  (-0.10, 0.08) |
|  | Model 4 | -0.06 (-0.13, 0.01) | -0.05  (-0.15, 0.05) | -0.12 (-0.23, -0.00) | 0.08 (-0.04, 0.19) | -0.01^$$^  (-0.09, 0.07) |
| Age 36-60  (ref for interaction) | Model 3 | -0.19  (-0.27, -0.11) | -0.12  (-0.21, -0.03) | -0.14  (-0.28, -0.01) | 0.15  (0.02, 0.28) | -0.23  (-0.36, -0.11) |
|  | Model 4 | -0.05  (-0.10, -0.01) | -0.08 (-0.17, 0.01) | -0.13 (-0.26, 0.00) | 0.09 (-0.03, 0.22) | -0.20  (-0.32, -0.08) |
| Fruit and vegetable intake | | | | | | |
| All ages  (11-60 years) | Model 3 | -0.11  (-0.17, -0.05) | -0.08  (-0.14, -0.02) | -0.10  (-0.19, -0.01) | 0.10  (0.04, 0.17) | -0.11  (-0.17, -0.04) |
|  | Model 4 | -0.07  (-0.10, -0.04) | -0.07  (-0.13, -0.01) | -0.10 (-0.19, -0.01) | 0.09  (0.02, 0.15) | -0.10 (-0.16, -0.03) |
| Age 11-18 | Model 3 | -0.04  (-0.15, 0.07) | -0.00  (-0.11, 0.10) | 0.02  (-0.06, 0.10) | -0.03^$^  (-0.14, 0.08) | 0.07^$$^  (-0.01, 0.15) |
|  | Model 4 | 0.00  (-0.07, 0.08) | -0.01  (-0.09, 0.11) | 0.02 (-0.06, 0.11) | -0.04  (-0.15, 0.06) | 0.08^$$^  (0.01, 0.16) |
| Age 19-35 | Model 3 | -0.05  (-0.17, 0.08) | -0.06  (-0.17, 0.04) | -0.12  (-0.21, -0.02) | 0.06  (-0.04, 0.16) | -0.04  (-0.11, 0.03) |
|  | Model 4 | -0.04  (-0.10, 0.03) | -0.05  (-0.16, 0.05) | -0.11 (-0.21, -0.02) | 0.04 (-0.04, 0.14) | -0.04  (-0.10, 0.03) |
| Age 36-60  (ref for interaction) | Model 3 | -0.13  (-0.21, -0.05) | -0.12  (-0.21, -0.03) | -0.09  (-0.24, 0.06) | 0.12  (0.02, 0.22) | -0.17  (-0.28, -0.05) |
|  | Model 4 | -0.04  (-0.08, -0.00) | -0.09  (-0.17, -0.01) | -0.08 (-0.22, 0.07) | 0.09  (-0.00, 0.18) | -0.14  (-0.26, -0.03) |
| F&V biomarker score | | | | | | |
| All ages | Model 3 | -0.28 (-0.34, -0.22) | -0.18 (-0.23, -0.12) | -0.08  (-0.14, -0.03) | 0.29 (0.22, 0.35) | -0.12 (-0.19, -0.05) |
|  | Model 4 | -0.09  (-0.13, -0.04) | -0.12  (-0.18, -0.06) | -0.07  (-0.12, -0.01) | 0.23  (0.16, 0.29) | -0.08  (-0.15, -0.01) |
| Age 11-18 | Model 3 | -0.20  (-0.31, -0.08) | -0.10 (-0.20, -0.01) | 0.04^$^  (-0.06, 0.14) | 0.21 (0.11, 0.31) | -0.02^$$^ (-0.09, 0.05) |
|  | Model 4 | -0.00  (-0.10, 0.09) | -0.04  (-0.14, 0.06) | 0.06^$^  (-0.04, 0.16) | 0.14  (0.04, 0.25) | 0.03^$$^  (-0.05, 0.10) |
| Age 19-35 | Model 3 | -0.30  (-0.44, -0.16) | -0.16  (-0.26, -0.06) | -0.04  (-0.11, 0.03) | 0.25 (0.14, 0.37) | -0.04^$^ (-0.15, 0.06) |
|  | Model 4 | -0.09  (-0.17, -0.01) | -0.10  (-0.20, 0.01) | -0.02  (-0.09, 0.05) | 0.19  (0.07, 0.31) | 0.00^$^  (-0.09, 0.10) |
| Age 36-60  (ref for interaction) | Model 3 | -0.26  (-0.33, -0.19) | -0.21  (-0.29, -0.12) | -0.12  (-0.21, -0.03) | 0.32 (0.24, 0.40) | -0.19 (-0.28, -0.09) |
|  | Model 4 | -0.05  (-0.10, -0.01) | -0.14  (-0.22, -0.06) | -0.10  (-0.18, -0.01) | 0.25  (0.17, 0.33) | -0.14  (-0.24, -0.03) |

Model 3 was adjusted for social class, smoking status, hours of sleep, taking dietary supplements and having a long-term health condition, and age-group specific covariates were derived from a model including interaction between exposure and age group. Model 4 is as model 3 but additionally adjusted for BMI z-score. Interactions with age group: $ indicates significant interaction between diet exposure and age category, compared to the reference age category (36-60 years). ^$$$^ p<0.001, ^$$^p<0.01, ^$^p<0.05.
